# Computed Tomographic Assessment of Individual Paranasal Sinus Compartment and Nasal Conchal Bulla Involvement in 300 Cases of Equine Sinonasal Disease

**DOI:** 10.3389/fvets.2020.580356

**Published:** 2020-10-27

**Authors:** Padraic M. Dixon, Tim P. Barnett, Rhiannon E. Morgan, Richard J. M. Reardon

**Affiliations:** ^1^The Royal (Dick) School of Veterinary Studies and The Roslin Institute, The University of Edinburgh, Edinburgh, United Kingdom; ^2^Rossdales Equine Hospital, Exning, United Kingdom; ^3^The Royal Veterinary College, Hatfield, United Kingdom

**Keywords:** equine, equine sinonasal imaging, equine sinus disease, equine nasal conchal bulla disease, sinus compartment involvement

## Abstract

**Background:** Computed tomographic (CT) imaging has allowed new anatomical studies and detailed clinical imaging of the complex, overlapping equine sinonasal structures. Despite the widespread use of CT, no study has specifically identified which compartments are most commonly affected with sinus disorders. CT has also shown the presence of intercurrent, ipsilateral nasal disorders, especially infection of the nasal conchal bullae (NCB) in many cases of sinus disease, but the frequency of intercurrent NCB infections has not been reported.

**Objectives:** To identify which sinus compartments are most commonly affected in horses with clinical sinus disorders and to record the prevalence of NCB involvement in such cases.

**Study Design:** Retrospective examination of CT images of horses with confirmed unilateral sinus disease.

**Methods:** The CT images of 300 horses, from three different equine hospitals with clinically confirmed sinus disease [mainly dental (53%) and primary sinusitis (25.7%)] were retrospectively examined to determine which sinus compartments and NCBs were affected.

**Results:** The rostral, more dependent sinus compartments were most commonly involved, i.e., the rostral maxillary sinus in 284/300 (94.7% affected) and the ventral conchal sinus (87% affected). The caudal maxillary sinus (65.3%), dorsal conchal sinus (52.7%), frontal sinus (26%), ethmoidal sinus (32%) and sphenopalatine sinus (28.7%) were less commonly affected. There was infection or destruction of the ipsilateral NCBs in 56% of horses with sinus disorders, including the ventral NCBs in 42.3%, dorsal NCBs in 29% and both NCBs in 18% of cases.

**Main Limitations:** The horses with sinonasal disease that underwent head CT imaging include more problematic cases and horses of high value, rather than the general horse population.

**Conclusions:** The more dependant (i.e., the RMS and VCS) sinus compartments are most commonly involved in sinus disorders, with the RMS involved in nearly every case. The more dorsally located sinuses (i.e., caudal group) are less commonly involved. Many horses with sinus disease also have disorders of their nasal conchal bullae and so the term *sinonasal disease* seems appropriate for these disorders.

## Introduction

Sinus disorders are important diseases in horses due to their frequent refractory nature. They can have multiple causes including cheek teeth apical infection, benign and malignant space-occupying intra-sinus growths, trauma, oro-maxillary fistulae and mycotic infections (1–8). In the absence of any identifiable underlying cause for sinus disorder, the remainder by default, are termed primary sinusitis (1, 2). Radiography can provide a limited amount of diagnostic information for these cases due to the complexity of the overlapping sinonasal structures that additionally, become distorted when diseased (9). The use of computed tomography (CT) has had a major impact on the diagnosis of equine sinonasal disorders, allowing detailed imaging of these complex anatomical structures in multiple planes without the presence of overlapping structures (10–16). In particular CT imaging has allowed accurate identification of cheek teeth periodontal, apical and endodontic disease (12, 15, 17–19).

The use of CT has also allowed three-dimensional anatomical studies to be performed that have significantly changed our understanding of sino-nasal and dental anatomy (20–24). In addition to helping diagnose the causes of sinus diseases and identify which compartments are involved, to allow more targeted surgical treatment, CT imaging has also clearly shown the presence of concurrent nasal disorders in many horses with sinus disorders (11, 25, 26). These include the presence of ipsilateral NCB infections in addition to nasal mucosal swelling and the presence of intranasal sequestrae, inspissated exudate, and sino-nasal fistulae (11, 25–27), some of which can be seen on nasal endoscopy (3–5, 26). The recent identification of ongoing nasal disorders including NCB empyema explains why some horses with sinus disorders do not respond to apparently effective treatment of the sinus disorder (5, 25, 26).

**Figure 1:**
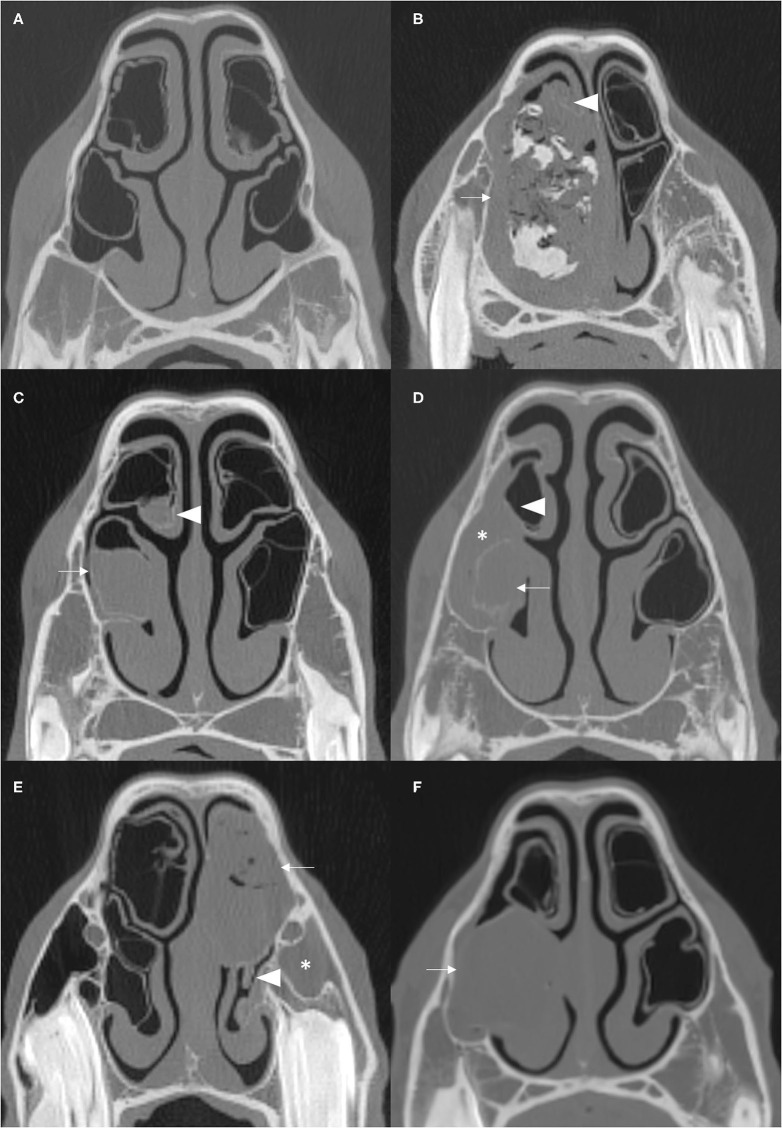
Transverse CT images of normal NCBs and of various types of NCB empyema. **(A)** Normal NCBs. **(B)** Mixed hyper- and hypoattenuation within a distended VCB, representing mineralisation and gas, respectively, within soft tissue attenuating material (arrow). There is compression and severe damage of the ipsilateral DCB (arrowhead) (mineralised nasal conchae found on histology—disorder of 10 years clinical duration). **(C)** Soft tissue/fluid attenuating material fills most of VCB (arrow) and the ventral aspect of DCB (arrowhead). **(D)** Soft tissue/fluid attenuating material fills the entire VCB (arrow), which has a thickening of the bony concha and has surrounding soft tissue/fluid attenuating material flowing from the ipsilateral sinuses (asterisk) and mild damage to the ipsilateral DCB (arrowhead). **(E)** The DCB is partially filled with material of mixed soft tissue and gas attenuation, reflecting inspissated purulent exudate (arrow). There is moderate damage of the ipsilateral VCB (arrowhead) and ipsilateral sinus empyema (asterisk). **(F)** The VCB is distended with homogenous soft tissue/fluid attenuating material (arrow). All CT images were reconstructed using using a bone filter (Window Level 800 HU, Window Width 2,800 HU). The right side of the patient is on the left side of the image. The transverse images **(A–C)** are at the level of the Triadan 08 maxillary cheek teeth, **(D,F)** at the level of the Triadan 07s and **(E)** is level with the distal (caudal) aspect of Triadan 08s.

**Figure 2:**
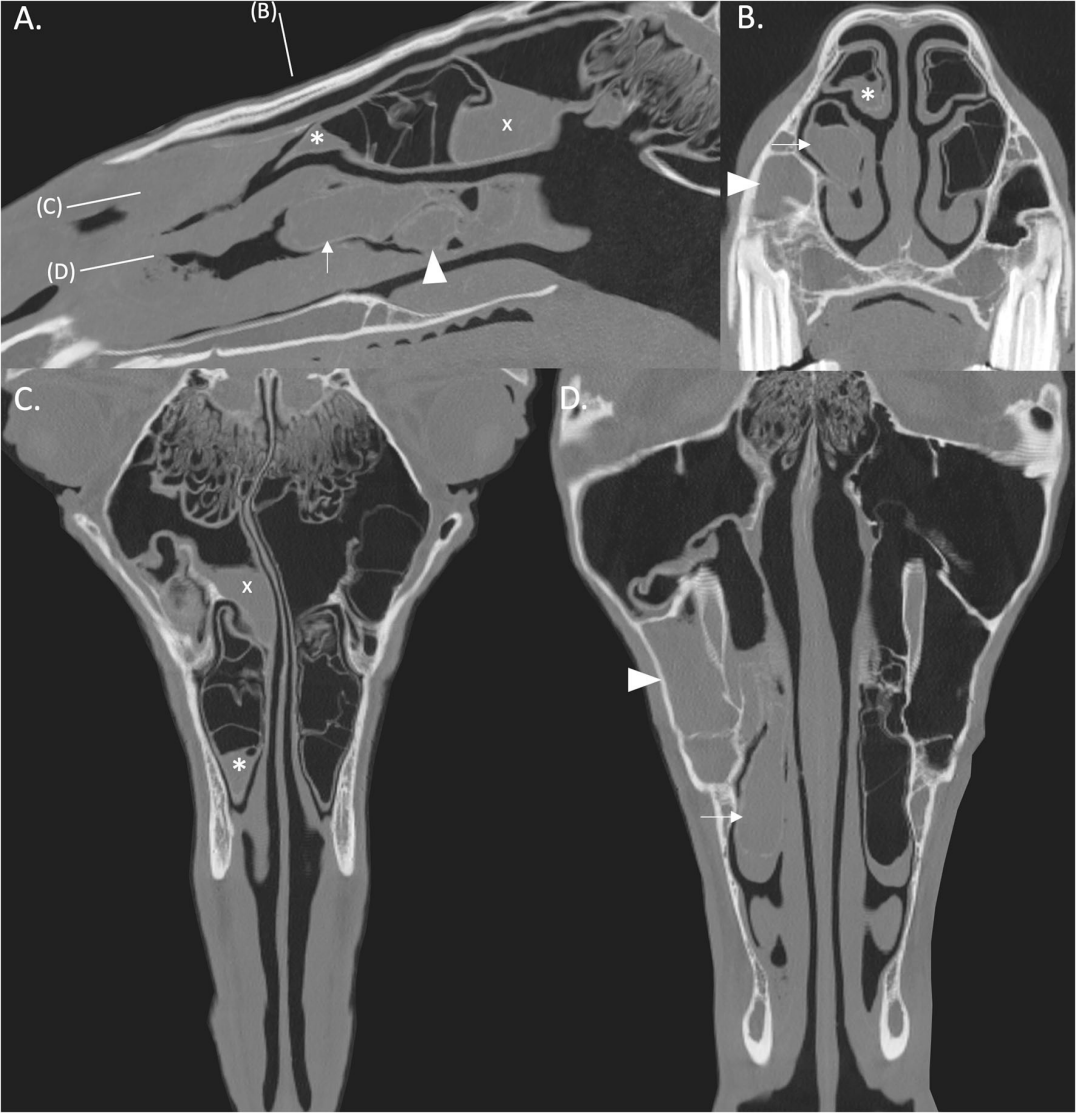
**(A)** Right parasagittal CT reconstruction with lines representing the locations of images (B–D). **(B)** Transverse CT image. **(C,D)** Dorsal CT reconstruction at the level of the DCB and VCB, respectively, the rostral aspect is toward the bottom of the image. There is empyema of the VCB (arrow) with ipsilateral sinusitis of the rostral (arrowhead) and caudal (x) paranasal sinus compartments. There is thickening of the mucosa of the rostral aspect of the right DCB (asterisk). All CT images are displayed using a bone filter (Window Level 800 HU, Window Width 2,800 HU). The right side of the patient is on the left side of the image.

**Figure 3:**
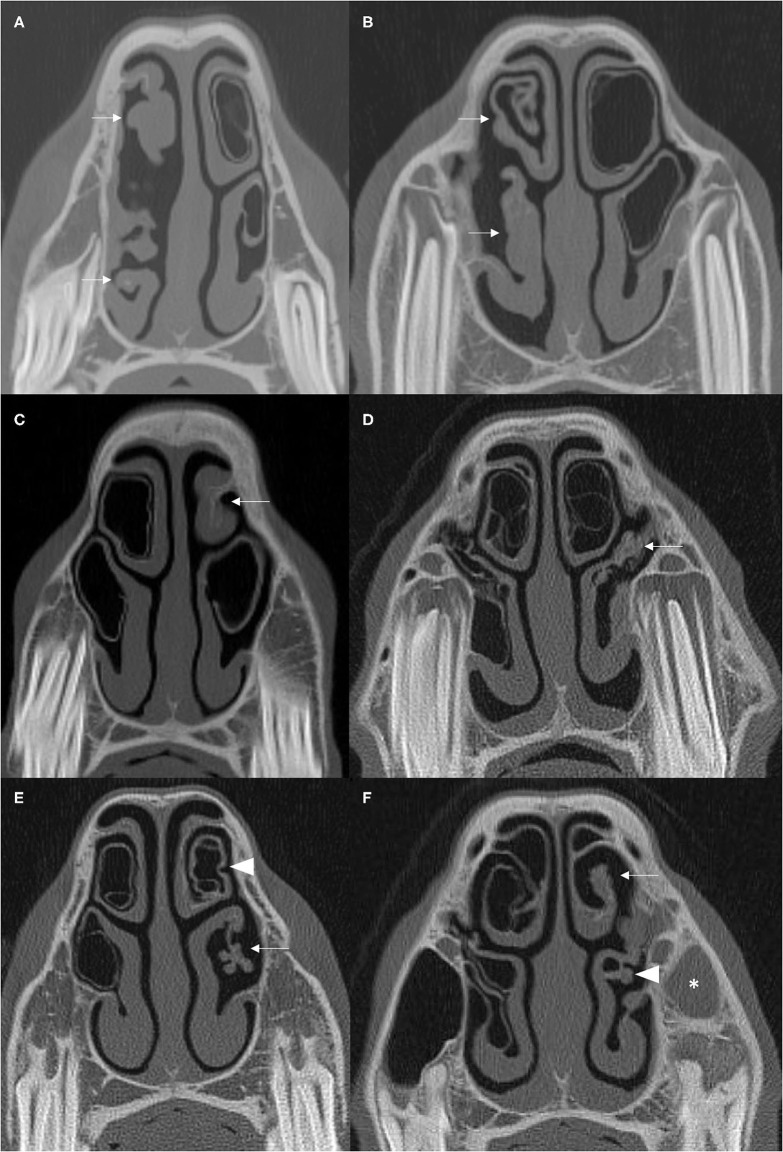
**(A,B)** Transverse CT images of two cases with moderate to severe damage of ipsilateral DCBs and VCBs (arrows) with distortion of the adjacent nasal concha. **(C)** The left DCB is not present (arrow) and there is contraction and thickening of the remaining adjacent nasal concha. **(D)** There is loss of the left VCB (arrow) with flattening and irregular thickening of the surrounding ventral nasal concha. **(E)** There is loss of the left VCB (arrow) with distortion and atrophy of the lateral aspect of the surrounding ventral concha. The walls of the ipsilateral DCB is hyper-attenuated and has a scalloped appearance (arrowhead). **(F)** There is loss of the DCB (arrow) and distortion and thickening of the adjacent concha and loss of identifiable structure in the VCB (arrowhead). There is soft tissue/fluid attenuation filling the left rostral maxillary sinus consistent with ipsilateral sinusitis (asterisk). All CT images are displayed using a bone filter (Window Level 800 HU, Window Width 2,800 HU). The right side of the patient is on the left side of the image. Transverse images **(A,E)** are at the level of the Triadan 07 maxillary cheek teeth, **(B–D)** at the level of the Triadan 08s and **(F)** is level with the distal (caudal) aspect of the Triadan 08s.

**Figure 4:**
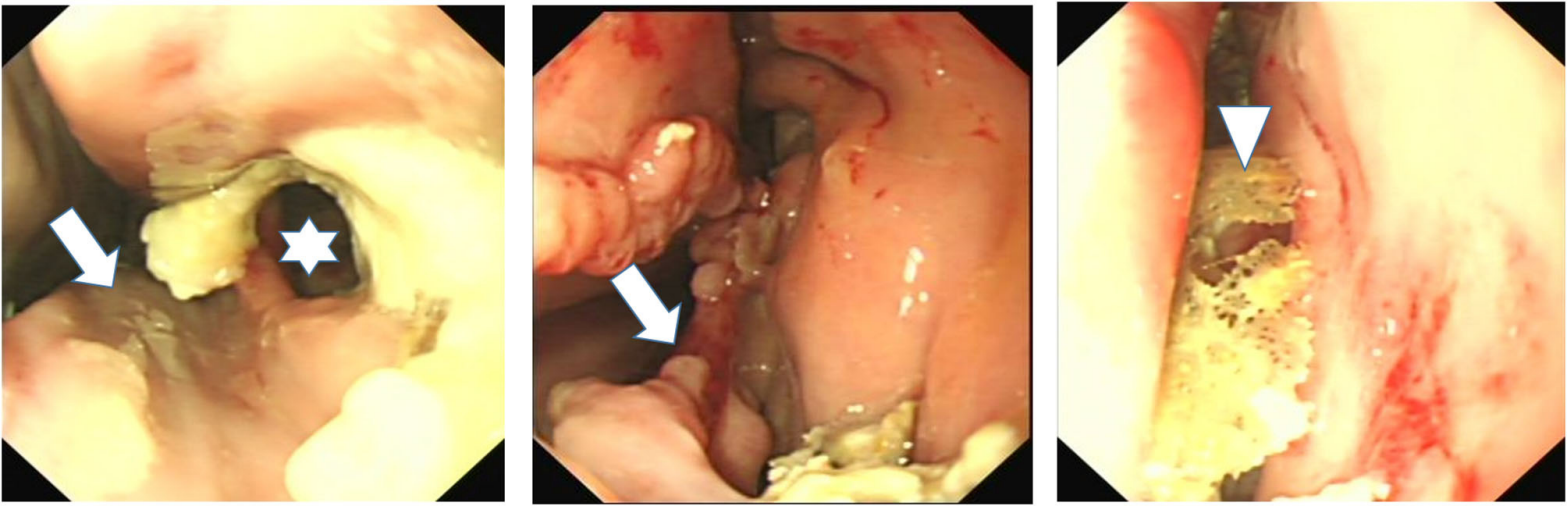
Nasal endoscopy image of the caudal aspect of the middle meatus of horses with ipsilateral sinus and nasal conchal bulla disease. Left image shows loss of VCB and of caudal aspect of ventral concha (arrow) and a fistula into VCS (star) surrounded by inspissated exudate. Middle image also shows loss of VCB and distortion of adjacent ventral concha with inspissated exudate at VCB site. The right image shows sequestered conchal bone lying in middle meatus after VCB infection and sequestration of its bony wall.

CT imaging can readily identify NCBs that are filled with fluid attenuating material, with or without regions of hypoattenuation (indicative of liquid and inspissated exudate) (Figures 1, 2). In other horses CT has identified gross disruption or even absence of the ipsilateral NCBs usually with distortion or local loss of the adjacent nasal concha (Figure 3). These changes appear to be advanced stages of NCB infection with their destruction or loss, along with local nasal conchal damage. Endoscopy of some such cases shows the presence of intranasal inspissated exudate and thin conchal sequestra at the caudal aspect of the middle meatus (Figure 4). However, it remains possible that absent or distorted NCBs and local nasal conchal distortion without the presence of exudate, could be developmental abnormalities or due to disorders other than ipsilateral sinus disease. That being the case, these changes should be bilaterally present in the nasal cavities in equal proportions.

Despite the importance of equine sinonasal disease, very limited information is available on which sinus compartments are most commonly involved in sinusitis. A study of 200 sinus disease cases where compartment involvement was determined by radiography and surgical exploration found the caudal maxillary (CMS), RMS and VCS compartments to be most commonly involved (3). In contrast, a recent CT study of 28 horses with sinus disorders found the RMS to be the most commonly involved compartment (was involved in all horses). No large study has objectively documented which sinus compartments are most commonly involved in disease.

The aim of this study was to retrospectively examine CT images from horses with sinoscopically and surgically confirmed unilateral sinus disease to identify which sinus compartments were affected and also to examine how many of these cases had empyema, destruction or loss of the ipsilateral NCBs. The causes of equine sinus disorders vary between different studies (1–8), likely due to different caseloads in different clinics and different geographical areas. In order to obtain more representative results in the current study, the retrospective examinations were performed using cases from three different clinics.

## Materials and Methods

The studies were performed in three clinics with different caseloads in different areas of the UK, including the Equine Hospitals of The Royal (Dick) School of Veterinary Studies (RDSVS), Edinburgh and of the Royal Veterinary College (RVC), London and Rossdales Equine Hospital (Rossdales), Newmarket. In each clinic, the CT images (all performed under standing sedation) and the clinical records of 100 recent, consecutive cases of clinically confirmed unilateral sinus disease were retrieved and re-examined by one author from each institution. Re-examination of these CT images were specifically performed to detect NCB and sinus compartment involvement using the pre-agreed criteria described later. A consensus was reached on images with ambivalent findings.

### Cause of Sinusitis

The etiology of each case of sinus disease as determined by their initial CT imaging, clinical, nasal endoscopic and sinoscopic examinations, surgical findings and by response to treatment was obtained from the clinical records. These included subacute (<2 months duration) and chronic (>2 months duration) primary sinusitis; dental sinusitis, sinus cysts; mycotic sinusitis; intra-sinus progressive ethmoid haematoma (PEH), sinus trauma; oro-maxillary fistula and sinus neoplasia and are presented in Table 1.

**Table 1:** Cause of sinus disease in 100 horses at each of three different centers.

**Cause of Sinusitis**	**RDSVS**	**RVC**	**Rossdales**	**Combined**
Subacute primary sinusitis	4%	14%	14%	10.7%
Chronic primary sinusitis	20%	10%	15%	15%
Dental Sinusitis	63%	49%	47%	53%
Sinus Cyst	8%	10%	7%	8.3%
Mycotic sinusitis	3%	3%	0%	2%
Progressive ethmoid haematoma	0%	1%	2%	1%
Traumatic Sinusitis	1%	6%	2%	3%
Oro-maxillary fistula	1%	5%	0%	2%
Sinus neoplasia	0%	2%	12%	4.7%

*RDSVS, University of Edinburgh; RVC, University of London*.

### CT Imaging

At the R(D)SVS, head CT images were obtained using a Siemens Somaton Volume Zoom 4 slice or a Siemens Definition AS 64-slice (Siemens, Munich, Germany) in a helical scan mode using a 512 × 512 Matrix, 120 Kv, 300 mA, at a slice thickness of 1.5 mm.

At the RVC, head CT images were obtained using a 16-slice multi-detector CT scanner (GE Lightspeed Pro 16, GE Medical Systems, Berkshire, UK) using 120 kV, 200 mAs, 1.25 mm slice thickness with an inter-slice interval of 1.25 mm. Images were reconstructed using both a bone and soft tissue algorithm in a 512 × 512 matrix.

At Rossdales, head CT images were obtained using a 16-slice multi-detector CT scanner (GE Lightspeed Pro 16, GE Medical Systems, Berkshire, UK) using 120 kV, 200 mAs, 0.625 mm slice thickness. Images were reconstructed using both a bone and soft tissue algorithm in a 512 × 512 matrix.

At all institutions the images were re-examined by the authors using Horos™ (Horos Project) software with the axes of the scans adjusted to obtain perpendicular transverse sections of the head for consistent measurements. Bone windows were used to review the images at a window width (WW) of 4000 Hounsfield Unit (HU) and window level (WL) of 1000 (HU).

### Individual Sinus Compartment Examinations

The CT images were examined for the presence of inflammation of individual sinus compartments as adjudged by the presence of fluid/soft tissue attenuation, i.e., from mucosal thickening and/or of accumulated exudate in their lumina. The conjoined dorsal conchal sinus (DCS) and the frontal sinus (FS) are usually considered as the single conchofrontal sinus (CFS) compartment. However, because long-term observations of clinical cases showed an apparent disparity between the involvements of the DCS and FS in sinus disease, inflammation of these compartments were recorded separately in this study. Frequency of individual sinus compartment involvement were compared between cases with dental and non-dental sinusitis using Chi-Squared tests in RStudio™, significance was set at *P* < 0.05.

### NCB Examinations

All cases were examined for alterations of the ipsilateral dorsal and ventral NCBs. NCBs with fluid or fluid and gas attenuation were readily identified as “NCB empyema” (Figures 1, 2). Other NCB changes including their deformation or absence, usually with various deformations of the adjacent ventral or dorsal nasal conchae (Figure 3) were also recorded as “NCB damage.” In case some of these absent or distorted NCBs and distorted adjacent nasal concha were developmental, or otherwise not related to concurrent ipsilateral sinus disease, all contralateral nasal cavities were also similarly examined for NCB changes.

**Figure 5:**
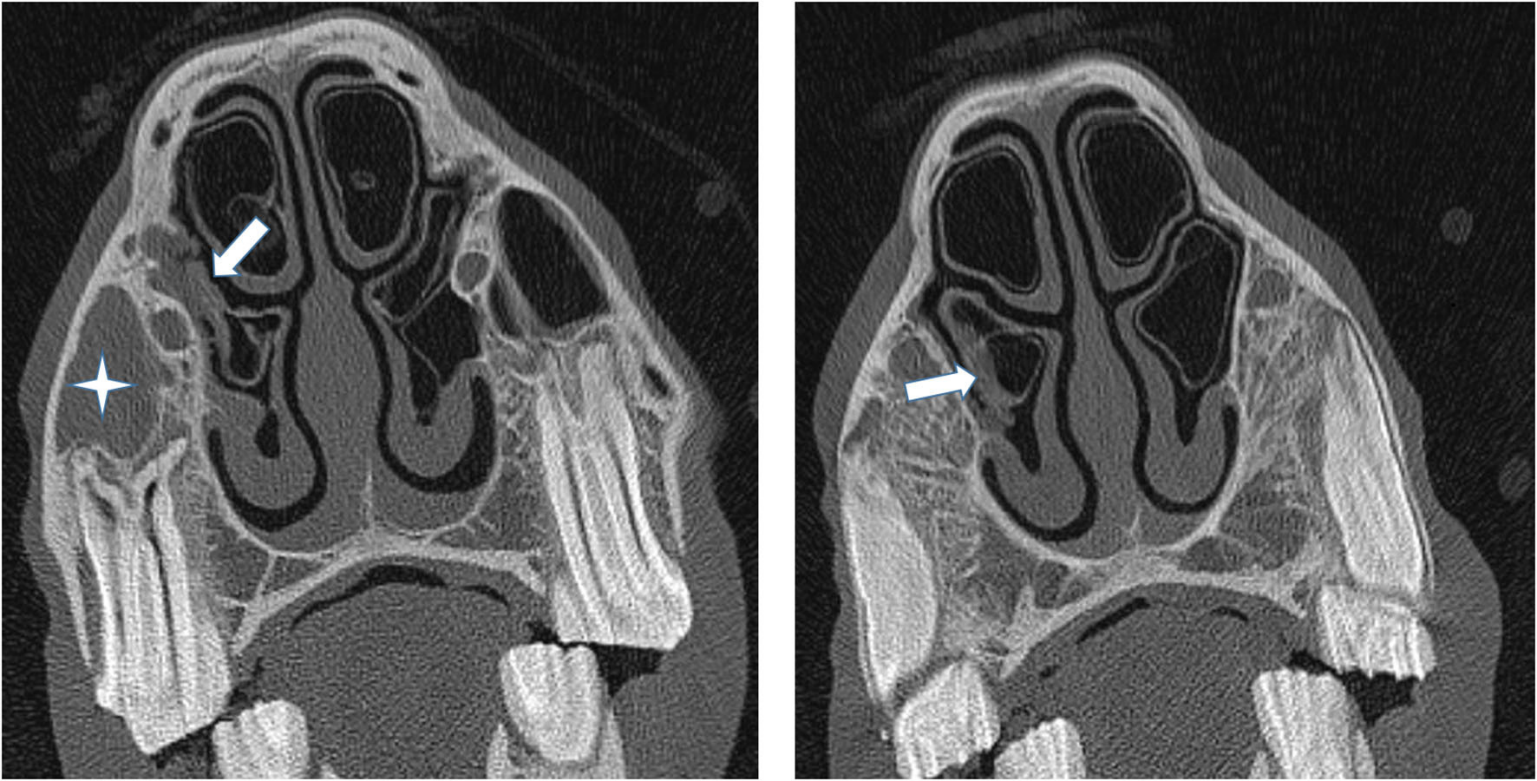
(Left) Transverse CT images of a horse suffering from empyema of the right RMS (star). The caudal aspect of the adjacent ventral concha is thickened and irregular (arrow) but more rostral sections (Right) showed the VCB not to contain exudate and it was classified as being normal.

It had been noted that the caudal aspects of NCBs adjacent to infected VCS and RMS compartments sometimes showed slight localized swelling of their walls, but the bullae did not contain any fluid attenuating material and were of normal appearance otherwise (Figure 5). These NCBs were classified as being normal. The NCBs adjacent to erupting cheek teeth in some young horses were compressed in a medial direction (23) and these changes were also regarded as a normal feature.

## Results

### Cause of the Sinus Disease

The causes of sinus disease in the 100 horses at the three centers and the combined values are presented in Table 1.

### Individual Sinus Compartment Involvement

**Figure 6:**
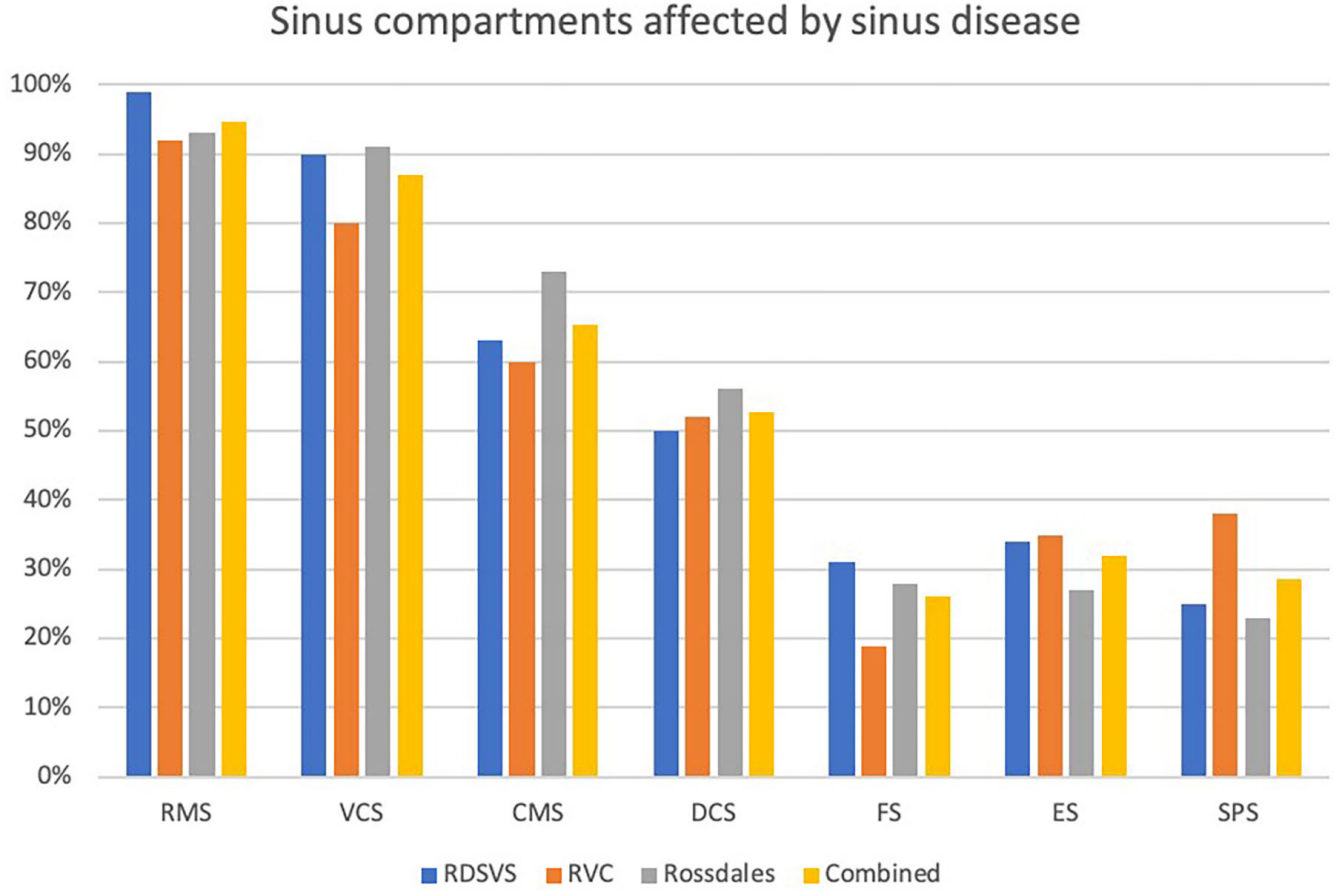
Histogram showing proportions of the different sinus compartments that were affected in 100 horses with sinus disease at each of three different centers. RMS, rostral maxillary sinus; VCS, ventral conchal sinus; CMS, caudal maxillary sinus; DCS, dorsal conchal sinus; FS, frontal sinus; ES, ethmoidal sinus (also termed the middle conchal sinus); SPS, sphenopalatine sinus (RDSVS, University of Edinburgh; RVC, University of London).

The proportions of individual sinus compartments affected by disease in each equine center are shown in Figure 6 and Table 2. There was a significantly higher frequency of RMS involvement in the Dental (98.2%) than the Non-dental (90.7%) sinusitis group [X^2^ (1) = 6.72, *P* = 0.009]; and significantly lower DCS and SPS involvement in the Dental (46.9 and 18.1%, respectively) than the Non-Dental (59.3 and 40.7%, respectively) sinusitis group [DCS X^2^ (1) = 4.13, *P* = 0.042 and SPS X^2^ (1) = 17.54, *P* < 0.001]. The frequency of other sinus compartment involvement did not differ significantly between the groups.

**Table 2:** Proportions of the different sinus compartments that were affected in 100 horses with sinus disease at each of three different centers.

**Compartment affected**	**Combined *N* = 300**	**Dental sinusitis *N* = 160**	**Non-dental sinusitis *N* = 140**	**X^**2**^ Dental vs. Non-Dental**
RMS	284 (94.7%)	157 (98.2%)	127 (90.7%)	X^2^ (1) = 6.72 ***P*** **=** **0.009**
VCS	261 (87%)	140 (87.5%)	121 (86.4%)	X^2^ (1) = 0.01 *P* = 0.918
CMS	196 (65.3%)	96 (60%)	100 (71.4%)	X^2^ (1) = 3.82 *P* = 0.051
DCS	158 (52.7%)	75 (46.9%)	83 (59.3%)	X^2^ (1) = 4.13 ***P*** **=** **0.042**
FS	78 (26%)	35 (21.9%)	43 (30.7%)	X^2^ (1) = 2.59 *P* = 0.108
ES	96 (32%)	44 (27.5%)	52 (37.1%)	X^2^ (1) = 2.76 *P* = 0.096
SPS	86 (28.7%)	29 (18.1%)	57 (40.7%)	X^2^ (1) = 17.54 ***P*** **<** **0.001**

*X^2^, Chi-square test with “Yates continuity correction,” significant P-values in bold; RMS, rostral maxillary sinus; VCS, ventral conchal sinus; CMS caudal maxillary sinus; DCS, dorsal conchal sinus; FS, frontal sinus; ES, ethmoidal sinus (also termed the middle conchal sinus); SPS, sphenopalatine sinus*.

### Changes to Ipsilateral and Contralateral NCBs

**Figure 7:**
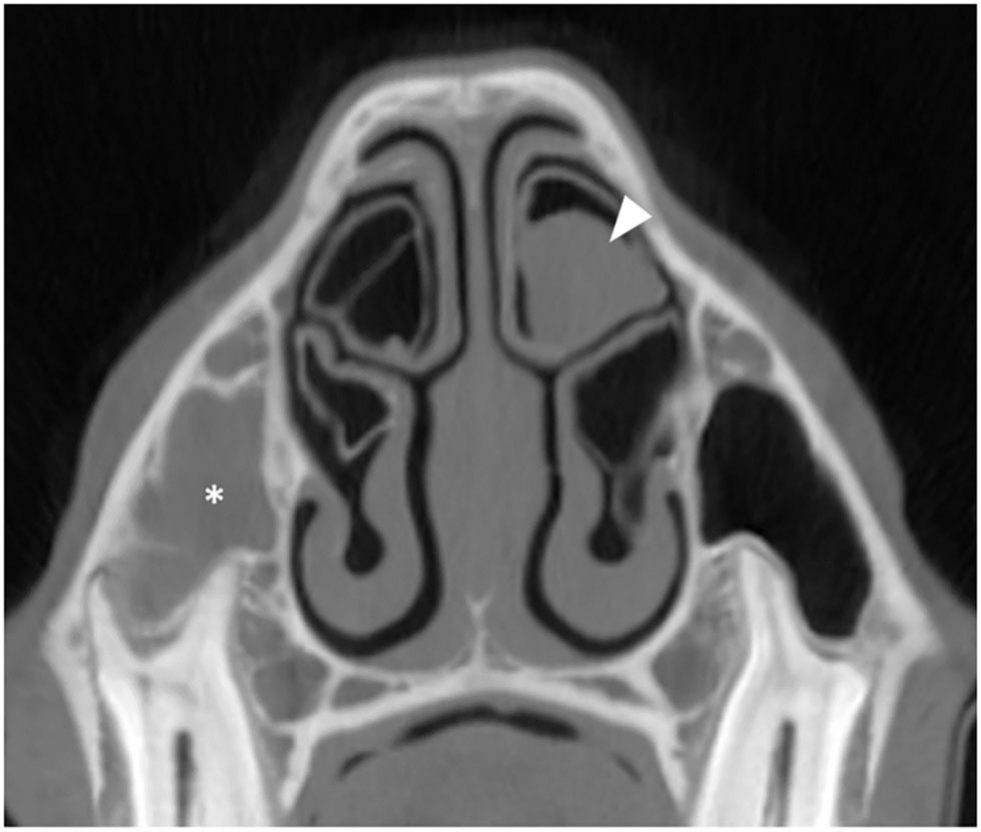
Transverse CT image of a horse suffering from right-sided sinus disease showing empyema of the right RMS (asterisk). The contralateral DCB contains soft tissue/fluid attenuating material indicative of empyema (arrowhead). All CT images are displayed using a bone filter (Window Level 800 HU, Window Width 2,800 HU). The right side of the patient is on the left side of the image.

The proportions of ipsilateral NCBs with empyema or “loss/damage” in each center are listed in Table 3. A consensus was reached on three ambivalent cases of possible NCB damage. Examination of the contralateral nasal cavity showed the presence of fluid attenuating material in DCBs (*n* = 4) (Figure 7) and VCBs (*n* = 2) and of loss or damage to VCBs (*n* = 2) and DCBs (*n* = 2), i.e., overall, 10/300 (3.3%) contralateral NCBs had abnormalities, including 6/300 (2%) with empyema. One horse suffering from a sinus cyst was found to have a fluid attenuating material in the region of its contralateral DCB that was later histologically shown to be a nasal cyst. Two horses had nasal abscesses adjacent to, but not involving their NCBs and these cases were classified as having normal NCBs.

**Table 3:** Proportions of 100 horses at each of three clinics with unilateral sinus disease that also had changes to their ipsilateral nasal conchal bullae (NCB) including their ventral conchal bullae (VCB) and dorsal conchal bullae (DCB).

**NCB involvement**	**RDSVS**	**RVC**	**Rossdales**	**Combined**
VCB empyema	24%	14%	22%	20%
VCB damaged	26%	24%	17%	22.3%
DCB empyema	15%	6%	23%	14.7%
DCB damaged	17%	16%	12%	14.3%
NCB involvement	58%	54%	56%	56%
No NCB involvement	42%	46%	44%	44%
Both NCB involved	24%	6%	18%	16%

*RDSVS, University of Edinburgh; RVC, University of London*.

## Discussion

### Cause of the Sinus Disease

The causes of sinus disease found in this study included 53% dental sinusits, 26% primary sinusitis; 8% sinus cyst with overall lower (<5%) proportions of mycotic sinusitis, PEH, trauma oromaxillary fistula and neoplasia (Table 1). The current findings are similar to a recent clinical audit of sinus disease, that found 45% of cases to be dental sinusitis, 36% primary sinusitis and 7% cysts (RJM Reardon Personal observations) but differ from an earlier clinical study at the same clinic that found primary sinusitis to be most commonly diagnosed cause of sinusitis (45% of cases), along with 20% dental sinusits and 13% sinus cyst (3). These changes in the pattern of sinus disease etiology may reflect changing referral clinic caseloads with higher proportion of primary sinusitis cases currently being treated in general practice, and with the more difficult, non-responsive cases, such as horses with dental sinusitis being referred. Additionally, not all cases of equine sinonasal disease receive CT imaging. Instead, the more complex and chronic cases and the more valuable horses are more likely to have such imaging and so the current findings may be biased in this respect.

### Sinus Compartment Involvement

This study found the two rostral compartments, i.e., the RMS (94.7% affected) and VCS (87%) to be most commonly affected compartments in all 300 horses with sinus disease (Table 2; Figure 6). There was decreasing involvement of the other compartments in a caudo-dorsal direction, from 65% involvment of the CMS to 28.7% of the SPS (Table 2; Figure 6). The Triadan 09s are the cheek teeth most commonly involved in dental sinusitis (1, 3, 4) and dental sinusitis was the most common cause of sinusitis in this study leading to empyema of the two rostral compartments with 98.2% RMS and 87.5% VCS involvement in cases of dental sinusitis. However, the RMS and VCS were affected in 90.7 and 86.4%, respectively, of non-dental sinusitis cases. The RMS and VCS are the most anatomically dependant compartments when the horse's head is in the resting or grazing positions and it is easy to understand how exudate would accumlate in them. Due to their dependant position all intra-sinus fluids, including normal mucus secretions as well as exudates have to be fully cleared by mucociliary clearance without the gravity assistance that occurs in the more dorsal sinus compartments. It is not surprising therefore that with sinus disease, some of this poorly draining exudate in the CMS and VCS later dehydrates and becomes inpissated (3, 4) leading to chronic or even permanent sinus disease.

The other caudal group of compartments, especially the three most dorsal compartments, i.e., the ES, FS and SPS are less dependant and were least commonly affected, but with higher involvement (non-significantly in 3/5 compartments) in non-dental sinusitis. These findings are somewhat similar to a CT study of 28 horses with sinus disease that found RMS involvement in 28/28 cases; CMS in 24/28; VCS and CFS in 23/28 and SPS in 18/28 (24). However, the current results differ from a study of 200 horses with sinus disorders, where CT imaging was not performed and where sinus compartment involvement was largely determined during surgical and sinoscopic exploration (3). In that study, the CMS (78% involvement) and RMS (61%) were most commonly affected, with lower involvement of the VCS (54%), CFS (48%) and combined ES and SPS (7%) (3). The difference between the current and that clinical study could be explained by inter-compartmental movement of blood and exudate during surgical exploration of the affected sinuses in the latter study (3), especially as some sinus osteotomies were performed in recumbent horses under general anesthesia.

There is no doubt that standing CT imaging is highly accurate and is the gold standard technique to identify sinus compartment involvement in cases of sinus disease. Consequently, the results of the current study are more accurate than clinical studies. This study again emphasizes the great importance of the two small, rostral VCS and RMS compartments in sinus disease and again highlights the enormous value of CT imaging in detecting sinus compartemnt involvement.

### Intercurrent Nasal Disorders

The equine nasal cavity is difficult to examine clinically and unless the middle meatus is carefully endoscopically examined (with a narrow endoscope, ideally <10 mm diameter), nasal endoscopy may not reveal much information. Partly for these reasons, equine nasal disease has been a neglected clinical area until recently. The use of CT has recently allowed new anatomical studies of this area, especially of the hitherto poorly described NCBs (24, 25), that in turn has allowed these structures now to be more clearly radiographically imaged (28). Most significantly, CT imaging has been proven invaluable in identifying intercurrent nasal disorders in horses with sinus disease, especially the presence of NCB infections (11, 24–26) and also sino-nasal fistulae (26).

The presence of infected NCB has been shown to be the cause of continuing clinical signs (unilateral purulent nasal discharge) in apparent non-responsive cases of sinus disease. Rarely NCB infections can cause chronic unilateral nasal discharge in the absence of ipsilateral sinus disease (25). Additionally, the recognition of this disorder has drawn clinical attention to this area and allowed nasal abnormalities other than infected NCBs including inspissated exudate, conchal sequestrae, mycotic plaques and sino-nasal fistulae to be identified on imaging and endoscopically (PM Dixon, unpublished observations).

No previous study appears to have reported the prevalence of ipsilateral NCB infection in horses with sinus disease. This study has shown 56% of horses with sinus disease to have changes in their ipsilateral NCBs, including NCB empyema in 34.7%, destructive changes with loss of the NCB and adjacent nasal conchal changes in 36.6% (16% of horses had one ipsilateral bulla with empyema and the other with destructive changes) (Table 3). NCB destructive changes are assumed to be caused by abscessation followed by rupture of these bullae. Nasal endoscopy has sometimes shown thin fragments of lace-like conchal bones that are possibly decalcified by chronic infection (not readily detectable on CT imaging) along with inspissated exudate adjacent to the NCB sites (RJM Reardon personal observations).

The pathogenesis of concurrent ipsilateral NCB infection in horses with sinus disease is likely to include their contamination by infectious exudate flowing from the adjacent sino-nasal drainage ostia, which can directly flow over the more commonly affected VCB. Additionally, horses with sinus disease invariably have swollen nasal mucosa (11) that could also disrupt normal NCB drainage and predispose to their infection. In horses with a sinonasal fistula (that are usually from the rostral aspect of the VCS into the middle meatus), it is very possible that the thin wall between the VCS and VCB could also be damaged leading to VCB empyema.

It was considered possible that the observed damaged or absent NCBs were not caused by the adjacent sinus disease but instead were a developmental abnormality or caused by some other non-sinus related mechanism such as mycotic rhinitis. Consequently, examination of the contralateral NCBs was performed in all cases. Surprisingly it showed 6/300 horses (2%) to have empyema of the contralateral NCBs that had not been clinically observed with only 4/300 cases (1.3%) having destructive changes. These findings suggest that the damaged or absent NCBs on the ipsilateral side to the sinus disease, that were in present in 110/300 (36.6%) of horses (27.5 times more commonly than on the contralateral side), were a sequel to the ipsilateral sinus disease.

The great clinical importance of intercurrent nasal disease in horses with sinus disorders is now well recognized and are currently treated appropriately, such as by draining NCBs and transendoscopic removal of sequestrae and inspissated exudate. Nevertheless, many long-term previous studies of sinus disease have shown that the majority of cases treated prior to the recognition of intercurrent nasal disease did resolve fully (8, 29, 30). However, this may because the treatments in these earlier studies usually involved sinusotomy and nasal fistulation, that along with prolonged high-volume sinus lavage, likely dislodged inspissated exudate and sequestrae from the NCB and middle meatus as well as from the sinuses, thus unknowingly treating any intercurrent nasal disorders. The finding that some horses have absence of some NCBs and distortion of adjacent nasal concha without any local exudate suggests that following infection and destruction of NCBs, with complete loss of NCB sequestra and inspissated exudate, that the clinical signs of NCB infection will also fully resolve.

## Conclusions

This multicenter CT study has shown dental sinusitis, primary sinusitis and sinus cysts to be the most common causes of equine sinus disease. The two rostral compartments, especially the RMS, are affected in nearly every case of sinus disease, with the CMS, CFS and other more dorsal compartments less commonly affected. The ipsilateral NCBs show evidence of current or past infection in 56% of horses with sinus disease. Increased attention should be given by imagers and clinicians to the high prevalence of intercurrent nasal disease in horses with sinus disorders.

## Data Availability Statement

The raw data supporting the conclusions of this article will be made available by the authors, without undue reservation.

## Author Contributions

PD contributed to study design and execution, data analysis and interpretation, and manuscript preparation. RM contributed to study execution, interpretation, and manuscript preparation. TB contributed to study execution, data analysis, and manuscript preparation. RR contributed to study design, data analysis and interpretation, and manuscript preparation. All authors contributed to the article and approved the submitted version.

## Conflict of Interest

The authors declare that the research was conducted in the absence of any commercial or financial relationships that could be construed as a potential conflict of interest.
